# Progress in Obstetrics

**Published:** 1898-04-09

**Authors:** 


### PROGRESS IN OBSTETRICS.

**Vaginal Hysterectomy.**—This operation in suitable cases has been very successful and has many advantages over the abdominal method. Freeland Barbour[1] gives a very complete description of Doyen's method, and an interesting historical sketch of complete extirpation of the uterus for cancer, the first operation recorded being performed in 1560 by Andreas A. Cruce. The operation was performed later by Sauter (1822), Blundell (1828), and Rècamier in 1829 introduced the ligature of the broad ligaments; while the clamp was suggested by Spencer Wells and its use amplified by Pean and Richelot in 1885 and 1886. The clamp may be used in two ways—first to prevent hæmorrhage temporarily; secondly, to check it definitely and finally, without the addition of any other method. Pean and Richelot adopt the former plan and Doyen the latter; the difference in the application is that in the first method the clamp is passed from below upwards and in the second from above downwards. The special feature of the clamps used by Doyen is that the blades are elastic, meeting at the points before the middle portions touch; the latter only meet on firm compression. Each blade is grooved longitudinally, so as to obtain a more powerful grip. The operation is performed by making a circular incision with the scissors through the vaginal mucous membrane around the cervix, the anterior incision being carried nearer to the external os, so as to avoid wounding the bladder. The pouch of Douglas is now opened with the scissors, and the separation of the bladder from the anterior wall of the uterus proceeded with. This accomplished, the anterior wall of the cervix and uterus is split longitudinally, and the incised edges seized with clamps, and the splitting continued until the utero-vesical pouch of peritoneum is reached. This is opened, and the opening enlarged by working through a pair of scissors and opening them. The fundus is then brought out to the front, and the broad ligament clamps applied from above downwards. A second smaller pair of clamps are now applied between the first pair and the uterus, and that organ removed by cutting between the two. The clamps are then turned downwards so that the points lie upwards and the broad ligaments are twisted on themselves. Iodoform gauze is now packed between the clamps and twisted around them to prevent pressure on the soft structures of the vagina. If it is desired to remove a uterus enlarged by fibroids by this operation, a V-shaped, instead of a longitudinal, incision is made in the anterior wall, and the included portion of tissue removed, and the process repeated until the fundus can be delivered. Severe pain is often caused after the operation by the pressure on the nerves included in the grip of the clamps, and cases of severe trophic reflex nervous disturbance occasionally occur.

Thorn[2] states that the average mortality after vaginal hysterectomy for carcinoma has fallen considerably. The lapses are due to portions of diseased tissue being left. About 43 per cent. relapse within the first two years after operation. Thorn is of opinion that total extirpation should be performed whenever the uterus is sufficiently movable; no operation should be performed if the uterus is fixed by carcinoma. Two per cent. of all patients attending a gynæcological clinique were affected with carcinoma out of 10,500 cases; 71 per cent. of the complicated cases relapsed within two years; but 90 per cent. of the uncomplicated cases survived the second year without relapsing, and about half of these may be looked upon as caused by vaginal extirpation. For improvement in the results Thorn is inclined to rely on early diagnosis and prompt operation rather than on new surgical procedures. The great advantages of the vaginal operation are the less amount of shock as compared with the operation per ventrum; the less liability to general peritoneal infection, and the more perfect drainage; with, in carcinoma of the uterus, the complete removal of the affected organ, and a smaller liability to intestinal adhesions.

**Abdominal Hysterectomy for Fibroids.**—The old method of operating by the abdominal route for fibroids with the use of the clamp extra abdominal treatment of the pedicle, has rightly fallen into disfavour, and is being replaced by various procedures, having for their object the ultra-abdominal but extra-peritoneal treatment of the stump, if any is left. One method is advocated by Bland Sutton,[3] who describes 28 cases in which he removed the uterus for myoma by supra-vaginal amputation after abdominal incision. Two deaths occurred in the series. The essential points in the technique are the treatment of the stump and the preservation in some cases of an ovary. After opening the abdomen and drawing up the uterus, the mesometria are ligatured and divided; after the attachment of the bladder is defined the peritoneum is incised on the anterior and posterior aspects of the uterus in such a way as to make the surfaces continuous with the openings in the mesometria, and the peritoneal flaps are raised from above downwards; the uterine arteries are then ligatured, and the uterus amputated above the ligatures; the anterior and posterior flaps of peritoneum are then united with silk sutures. In operating by this method he claims that the chance of damaging the ureters is greatly lessened. He considers there is always a risk of this accident in the operation of panhysterectomy, also that the preservation of an ovary where possible leads to a more rapid convalescence. He considered that this operation entirely obviated the necessity for the operation of öophorectomy.

Bland Sutton's paper was met with considerable criticism by the members of the Obstetrical Society present at the meeting when the paper was read. Many members considered that the operation of öophorectomy would still have its place as a method of treatment for fibroids, and that in many cases no mutilating operation was necessary, as it was often possible to carry the patient over the menopause by local treatment.

The operation of pure hysterectomy, or complete ablation of the uterus by combined vaginal and abdominal incision, has been successfully practised by several operators, and would appear to have certain advantages, such as freedom of drainage over supra-vaginal amputation; the danger of wounding the ureters, already alluded to by Bland Sutton, is not, however, remote, and necessitates great care in the ligation of the uterine arteries. This danger is increased by ligation of the broad ligaments in mass ligatures, which include much tissue, in addition to the vessels.

[1] Scottish Med. and Surg. Journal, Nov., 1897. [2] Lancet, Jan. 1st, 1898. [3] B. M. J., Nov. 13th, 1897.

